# An Unusual Case of Myonecrosis

**DOI:** 10.1155/2011/624020

**Published:** 2011-07-28

**Authors:** P. Mukhopadhyay, R. Barai, C. A. Philips, J. Ghosh, S. Saha

**Affiliations:** ^1^Department of General Medicine, Nilratan Sircar Medical College and Hospital, Kolkata 700014, India; ^2^Phani Kutir, Udaypur (South), 82 Olay Chandi Road, Kolkata 700049, India

## Abstract

Diabetic Myonecrosis is a rare complication of long-standing Diabetes Mellitus Type 1 and 2. The most likely affected areas are of proximal lower limbs, mostly the quadriceps muscle. The presenting features are myriad and a diagnostic conundrum for the physician. There has been previously mentioned, through few case reports, the classical presentation of diabetes-related muscle infarction. Here we present a patient of diabetic myonecrosis, in whom the initial presentation of diabetes mellitus was that of bilateral symmetric proximal upper limb predominant muscle infarction, which has never been reported before.

## 1. Introduction

Diabetic Myonecrosis is an enigmatic, rare complication of long-standing diabetes mellitus type 1 and 2. Most patients have associated retinopathy, nephropathy, or neuropathy. It commonly affects the lower limb muscles, predominantly the quadriceps. The pathogenesis of this condition is less understood for which more physicians are identifying this uncommon entity, since its first description in 1965 by Angervall and Stener [1]. The treatment for this particular manifestation of diabetes is watchful conservative management and strict glycemic control and in most cases produces a good outcome.

## 2. Case Report

A 58-year-old male patient, presented to our Emergency Department with features of bilateral proximal upper limb predominant muscle pain which started off as dull ache in character and over the course of 8 days, became excruciating in nature associated with swelling and restriction of movements at the shoulder joints. He was neither a known hypertensive nor a diabetic. There was no history of direct or indirect trauma; skin changes with any “over-the-counter” or herbal drug intake, apart from occasional acetaminophen (500 mg) tablets for pain. On further question, he admitted to have episodes of polyuria and polydipsia for a period of 2 years but considered these trivial. His mother and an elder sibling suffered from Diabetes Mellitus type 2 with good glycemic control on oral hypoglycemic agents.

**Figure 1:**
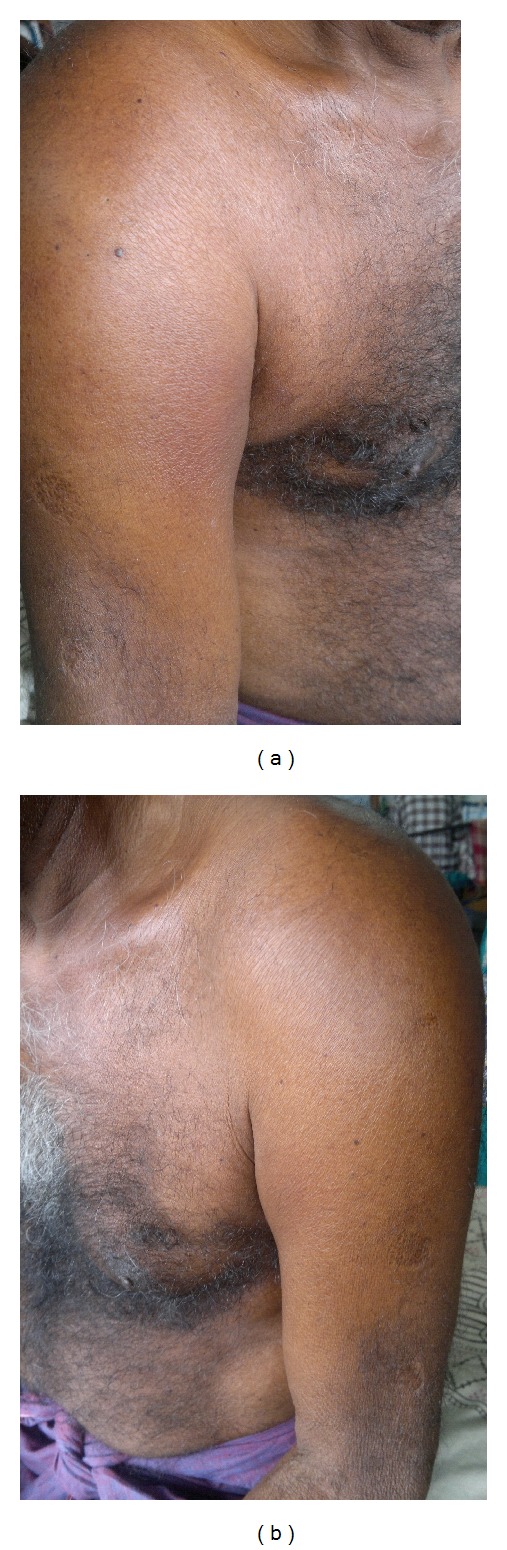
The muscles around the right and left shoulder joints showing features of swelling and mild erythema.

On examination there were local warmth, tenderness, and swelling around the shoulder joints and upper part of both arms associated with mild erythema, no induration, and no palpable crepitus (Figure 1). The patient was afebrile and normotensive with a Body Mass Index (the weight in kilograms divided by the square of height in meters) of 29.4. All the peripheral pulses were palpable. Deep tendon reflexes were present and normal in all four limbs. A fundoscopy revealed small microaneurysms of the retinal vessels with small blot hemorrhages. Other systems examination was noncontributory. The hemogram revealed hemoglobin levels to be 13 g/dl, a slightly raised total leucocyte count of 12.9 × 10^9^ (normal—3.8 to 9.8 × 10^9^/L) with a neutrophilic predominance (94%) in the differential and normal liver function and renal function tests. An initial random blood glucose on admission was 440 mg/dl and a subsequent fasting blood glucose and 2-hour postprandial blood glucose done the next day revealed values of 280 mg/dl and 308 mg/dl, respectively. The Hba1c was 10.1% (normal—less than 5.5%). The serum creatine kinase (CK) level was 80 U/L (normal—24 to 195 U/L) while serum lactic acid dehydrogenase (LD) was 168 U/L (normal—45 to 90 U/L). A spot urine for albumin to creatine ratio was 110 microgram albumin per milligram creatine (normal in male—30 to 300 microgram per milligram). His thyroid profile tests, serum uric acid, and electrolytes were well within normal limits. 

A Roentgenogram of the shoulder joints did not reveal any bony abnormalities, but there was evidence of mild soft tissue swelling. Ultrasonography around both shoulder joints and upper arms showed diffuse swelling of soft tissue of muscle compartments without any evidence of local abscess or muscle tumor and a subsequent Doppler study revealed absence of deep vein thrombosis.

**Figure 2:**
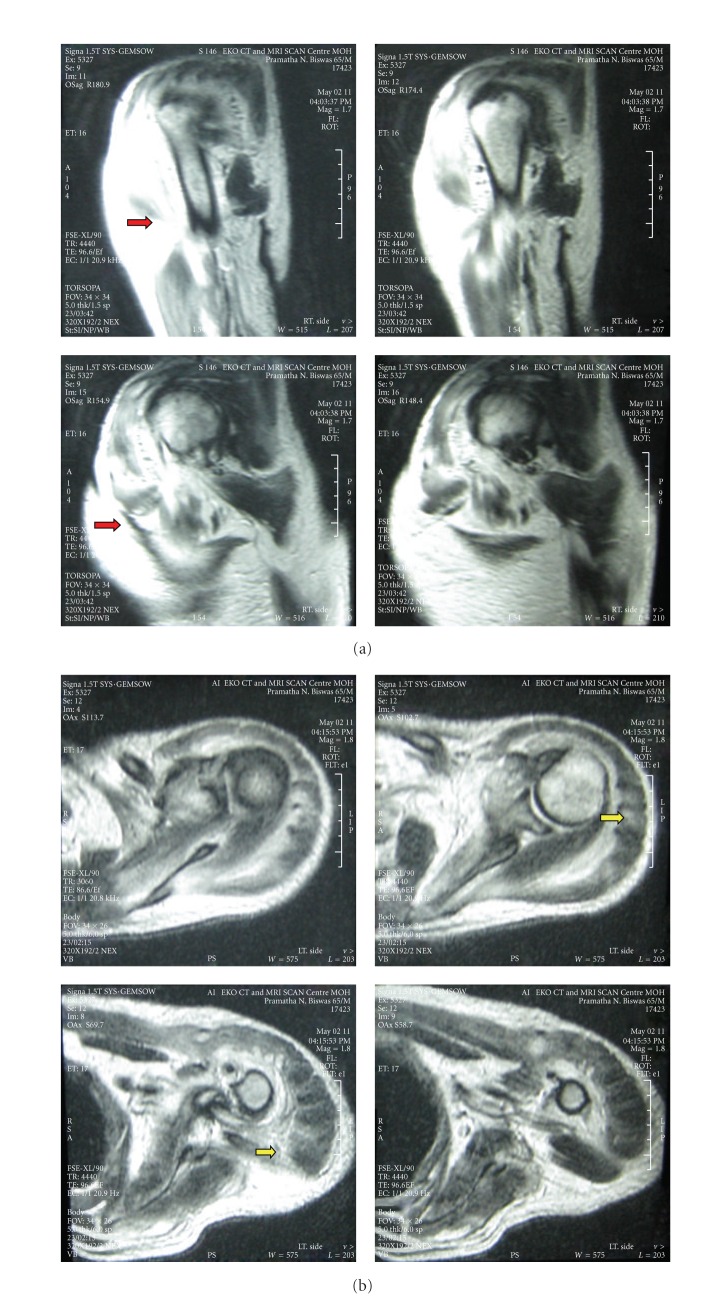
(a) Magnetic Resonance Imaging (T2W) of muscles around the right shoulder joint and right anterior chest region showing areas of increased signal intensities with areas of myositis, muscle swelling (red arrows), and subcutaneous edema. (b) Magnetic Resonance Imaging (T2W) of the muscles around the left shoulder joint revealing areas of hyperintensities suggestive of necrosis (yellow arrows) and inflammation of muscles with subcutaneous edema.

A Magnetic Resonance Imaging of the right and left upper limbs showed features of myositis, myoedema, and focal necrotic regions (greater on the left) and subcutaneous edema with increased signal intensities on T2-weighted images, involving deltoid, supraspinatus, and pectoralis muscles. There were also mild increased signal changes seen from subscapularis as well as teres muscle (Figures 2(a) and 2(b)). 

Considering the clinical and radiological findings, a diagnosis of Diabetic myonecrosis was made and the patient was started on analgesics, aggressive insulin therapy, and absolute bed rest. Within 4 weeks of admission, he showed signs and symptoms of improvement with decrement in pain with near normalization of his glycemic status. A repeat glycated hemoglobin level was 6.1 after a month and a half. 

## 3. Discussion

Diabetic myonecrosis or Diabetic muscle infarction as it is called is an uncommon complication of Diabetes Mellitus. Most patients have long-standing diabetes with or without extensive end organ damage, as a result of microvascular disease [2]. Severe diabetic microangiopathy has been proposed as the underlying mechanism that leads to spontaneous nongangrenous and focalized muscle infarction. Other proposed theories include coagulation-fibrinolysis derangement, hypoxia-reperfusion abnormalities, and recently, the role of anti-phospholipid antibodies [3].

Usual initial manifestations are acute or subacute onset of intense pain of involved muscles. There is a clear predilection for thigh muscles and the disease rarely presents as bilateral disease and further rarely involving the upper limb muscles. The most commonly affected muscles are quadriceps, hip adductors, and ham strings [4]. CK levels are generally normal, with a normal or slightly elevated LD levels. There may be associated leucocytosis. Upper limb involvement in diabetes mellitus is extremely rare. Very few reports have shown unique involvement of the unilateral arm muscles [5, 6]. Initial presentation of diabetes mellitus in a patient as diabetic myonecrosis has been described only once before by the classical lower limb predominant presentation [7]. Bilateral upper limb proximal muscle involvement has never been reported before. 

Muscle biopsy is the gold standard for diagnosis. But current studies and experience teach us that a muscle biopsy or surgical excision of the affected muscles will either prolong the disease in the individual or acutely exacerbate this condition. Usually MRI and clinical examination will suffice, in coming to a diagnosis of diabetic muscle infarction [8]. The differential diagnosis includes infections (myositis, cellulitis, abscess, necrotizing fasciitis, and osteomyelitis), trauma (hematoma, muscle rupture, myositis ossificans), vascular (deep vein thrombosis, compartment syndrome), tumors, inflammatory muscle diseases, and drug-related myositis (statin group) [9]. The short-term prognosis is generally good, but the long-term prognosis is complicated by recurrences in a previously affected muscle or muscles of the opposite limb. Nearly half of these recurrences occur within 2 months of initial presentation. Treatment includes adequate rest, analgesics, and good glycemic control. Some authors propose the use of antiplatelet therapy to treat the underlying micro-vasculopathy, but this is not a strict recommendation [10]. 

There has been only one case report from India on this condition previously [11].

Our case is unique in the sense that the initial presentation of Diabetes Mellitus was that of myonecrosis involving bilateral proximal muscles of the upper limb, mainly the deltoids and pectoralis muscles.
